# Real‐world risk factors of confirmed or probable COVID‐19 in Americans with diabetes: A prospective, community‐based study (iNPHORM)

**DOI:** 10.1002/edm2.342

**Published:** 2022-05-29

**Authors:** Alexandria Ratzki‐Leewing, Jason E. Black, Bridget L. Ryan, Stewart B. Harris

**Affiliations:** ^1^ Department of Epidemiology and Biostatistics, Schulich School of Medicine and Dentistry Western University London Ontario Canada; ^2^ Department of Family Medicine, Schulich School of Medicine and Dentistry Western University London Ontario Canada; ^3^ Department of Medicine, Schulich School of Medicine and Dentistry Western University London Ontario Canada

**Keywords:** COVID‐19, diabetes mellitus, type 1, diabetes mellitus, type 2

## Abstract

**Introduction:**

Americans with diabetes are clinically vulnerable to worse COVID‐19 outcomes; thus, insight into how to prevent infection is imperative. Using longitudinal, prospective data from the real‐world iNPHORM study, we identify the intrinsic and extrinsic risk factors of confirmed or probable COVID‐19 in people with type 1 or 2 diabetes.

**Methods:**

The iNPHORM study recruited 1206 Americans (18–90 years) with insulin‐ and/or secretagogue‐treated type 1 or 2 diabetes from a probability‐based internet panel. Online questionnaires (screener, baseline and 12 monthly follow‐ups) assessed COVID‐19 incidence and various plausible intrinsic and extrinsic factors. Multivariable Cox regression was used to model the rate of COVID‐19 (confirmed or probable). Risk factors were selected using a repeated backwards‐selection ‘voting’ procedure.

**Results:**

A sub‐sample of 817 iNPHORM participants (type 1 diabetes: 16.9%; age: 52.1 [SD: 14.2] years; female: 50.2%) was analysed between May 2020 and March 2021. During this period, 13.7% reported confirmed or probable COVID‐19. Age, body mass index, number of chronic comorbidities, most recent A1C, past severe hypoglycaemia, and employment status were selected in our final model. Body mass index ≥30 kg/m^2^ versus <30 kg/m^2^ (HR 1.63 [1.05; 2.52]_95% CI_), and increased number of comorbidities (HR 1.16 [1.05; 1.27]_95% CI_) independently predicted COVID‐19 incidence. Marginally significant effects were observed for overall A1C (*p* = .06) and employment status (*p* = .07).

**Conclusions:**

This is the first US‐based epidemiologic investigation to characterize community‐based COVID‐19 susceptibility in diabetes. Our results reveal specific and promising avenues to prevent COVID‐19 in this at‐risk population. ClinicalTrials.gov Identifier: NCT04219514.

## Graphical abstract

Americans with type 1 or 2 diabetes are especially vulnerable to severe COVID‐19 outcomes. Diabetes epidemiologic research on COVID‐19 susceptibility is required to better understand viral transmission, and to guide targeted prevention strategies. Leveraging longitudinal, prospective data from iNPHORM, we present the first primary analysis on the real‐world incidence, and time‐varying risk factors of COVID‐19 (confirmed or probable) in the general American population with diabetes. Our study offers an important roadmap for mitigating COVID‐19 in Americans with diabetes at highest risk of contraction. Diabetes‐specific COVID‐19 interventions should prioritize those reporting a BMI ≥ 30 kg/m^2^, comorbidity, A1Cs ≥9.1% and ≤7%, or part‐time employment.

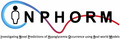

## 1 INTRODUCTION

Despite extensive vaccine rollouts, the United States (US) continues to report the highest numbers of confirmed COVID‐19 diagnoses and fatalities in the world.^1^ Type 1 and 2 diabetes ranks as the second most common underlying health condition among US cases,^2^ contributing to an elevated risk of severe outcomes.^3^ Even for the majority reporting very mild infection, diabetes has shown to exacerbate post COVID‐19 syndrome (i.e. ‘long COVID’),^4^ exposing an already high‐risk population to further medical, social, and economic challenges.^4^

Insight into the factors altering COVID‐19 susceptibility in diabetes is needed to understand viral transmission and control over time—especially as the virus becomes increasingly endemic. Hospital‐based studies have done well to quantify the burden and predictors of severe COVID‐19 morbidity and mortality in patients experiencing hyperglycaemia,^5^ helping usher improved antiviral and treatment protocols. However, what remains unknown is the overall epidemiology of COVID‐19 in the US public with diabetes, and strategies to prevent it.

Here, we analyse longitudinal, prospective data from the real‐world iNPHORM (Investigating Novel Predictions of Hypoglycemia Occurrence using Real‐world Models) study to ascertain the real‐world, time‐varying distribution and determinants of confirmed or probable COVID‐19 in people with diabetes. The results of this study will be instructive for future clinical and public health strategies aimed at mitigating COVID‐19 risk in one of America's most prevalent and vulnerable disease populations.^3^

## 2 MATERIALS AND METHODS

### 2.1 Study design

The current evaluation describes a sub‐analysis of the larger iNPHORM panel survey: a 1‐year prospective investigation of real‐world hypoglycaemia risk stratification in the US.^6^ Longitudinal self‐assessed data were examined to determine the incidence proportion and related intrinsic and extrinsic factors of infection with the SARS‐CoV‐2 virus leading to COVID‐19 between May 2020 and March 2021.

### 2.2 Participants and data collection

Individuals 18–90 years old, living in the US (≥1 year), with a self‐reported diagnosis of insulin and/or secretagogue‐treated type 1 or 2 diabetes (≥1 year) were eligible to enrol in the iNPHORM study; those involved in a concurrent intervention or pregnant (at screener or within year prior) were ineligible. Recruitment occurred across two sub‐panels (A and B) conveniently sampled from a representative (geodemographic, attitudinal, behavioural), probability‐based internet panel of Americans with diabetes (type 1 diabetes: *N* ≈ 10,000; type 2 diabetes: *N* ≈ 58,000). Enrolees were managed and hosted by Ipsos Interactive Services (IIS), a global leader in real‐world, patient‐centred survey conduct.

Sub‐panels A and B completed an online baseline questionnaire and up to 12 waves of follow‐up (monthly questionnaires) that were emailed automatically by IIS; the follow‐up schedule between panels was offset by 2 months (Sub‐panel A: February 2020 to January 2021; Sub‐panel B: April 2020 to March 2021). Participants were given 7 days to complete each follow‐up questionnaire using various internet‐equipped devices (e.g. computers, tablets and smartphones). All responses were synchronously stored on the IIS platform. Reminders and token incentives were distributed prospectively.

iNPHORM questionnaires (screener, baseline and follow‐ups) were produced in English and pretested/piloted before fielding. Questionnaires captured self‐assessed data on non‐clinical and clinical, intrinsic and extrinsic variables. In response to the escalating severity of the pandemic, follow‐ups were emended to include a COVID‐19 sub‐questionnaire starting in May 2020 (Sub‐panel A: Wave 3; Sub‐panel B: Wave 1). Further details on iNPHORM (ClinicalTrials.gov Identifier: NCT04219514) procedures and instruments are available elsewhere.^6^

### 2.3 COVID‐19 status

Structured items were disseminated to classify respondents as confirmed or probable COVID‐19 cases based on guidelines from the Centers for Disease Control and Prevention (April 2020).^7^ Confirmed cases were those who reported having been medically diagnosed with COVID‐19 by either ribonucleic acid or viral antigen assay. Probable cases were those who did not have a formal medical diagnosis but who reported (1) symptoms typical of COVID‐19 (e.g. a cough, difficulty breathing, fever [over 100 degrees Fahrenheit], sore throat, headache, tiredness, or muscle aches and pains) and (2) ≥1 form of epidemiological exposure (close contact with confirmed/suspected case or international travel). At each follow‐up, participants were asked to report on their COVID‐19 status since their last completed questionnaire.

### 2.4 Potential risk factors

A broad range of plausible intrinsic and extrinsic COVID‐19 risk factors were determined in consultation with the literature and diabetes experts. Information pertaining to these variables, including time‐varying characteristics, were collated between the screener, baseline and follow‐up questionnaires (Table S1). In this analysis, the following factors were evaluated:

#### 2.4.1 Intrinsic variables

Age (18–29, 30–49, and ≥ 50 years); sex assigned at birth; diabetes type; duration of diabetes; most recent A1C (≤53.0 mmol/L [≤7%], 54–64 mmol/L [7.1%–8%], 65–75 mmol/L [8.1%–9%], and ≥76 mmol/L [≥9.1%]); body mass index (BMI) (≥30 vs. <30 kg/m^2^); type of chronic comorbidity (bone disorder; cancer or human immunodeficiency virus; cardiovascular disease, stroke, hypertension; chronic kidney disease; gastrointestinal disease or chronic liver failure; mental health or eating disorder; neurological disorder or physical impairment; and respiratory condition); number of chronic comorbidities; number of diabetes complications; and past severe hypoglycaemia.

#### 2.4.2 Extrinsic variables

Geographic region; rurality; employment; highest level of education; health literacy; income and number of household members; health insurance; marital status; living arrangement; race (proxy for systemic marginalization); use of insulin and/or secretagogues; use of continuous glucose monitoring; frequency of healthcare visits for diabetes; and physical distancing.

### 2.5 Statistical analysis

We analysed May 2020 responders (Sub‐panel A: Wave 3 and Sub‐panel B: Wave 1) who, at that time, reported no current or previous COVID‐19 (confirmed or probable); and who completed at least one subsequent monthly questionnaire. Thus, in this study, follow‐up spanned 9 months for Sub‐panel A and 11 months for Sub‐panel B.

Sample characteristics, based on May 2020 responses, were summarized as frequencies and percentages for categorical variables, and as means and standard deviations (SD) or medians and interquartile ranges (IQR) for continuous variables. Incidence proportions were calculated for first reported COVID‐19 cases occurring between May 2020 and March 2021.

Multivariable Cox proportional hazards regression, accounting for time‐varying risk factors, was used to model the hazard rate of confirmed or probable COVID‐19. A cause‐specific hazard function adjusted for the competing effect of COVID‐19 vaccination.^8^ Ten imputed datasets were generated using multiple imputation by chained equations.^9^, ^10^, ^11^ Missing time‐varying risk factor data were imputed as separate terms for each follow‐up.

**FIGURE 1:**
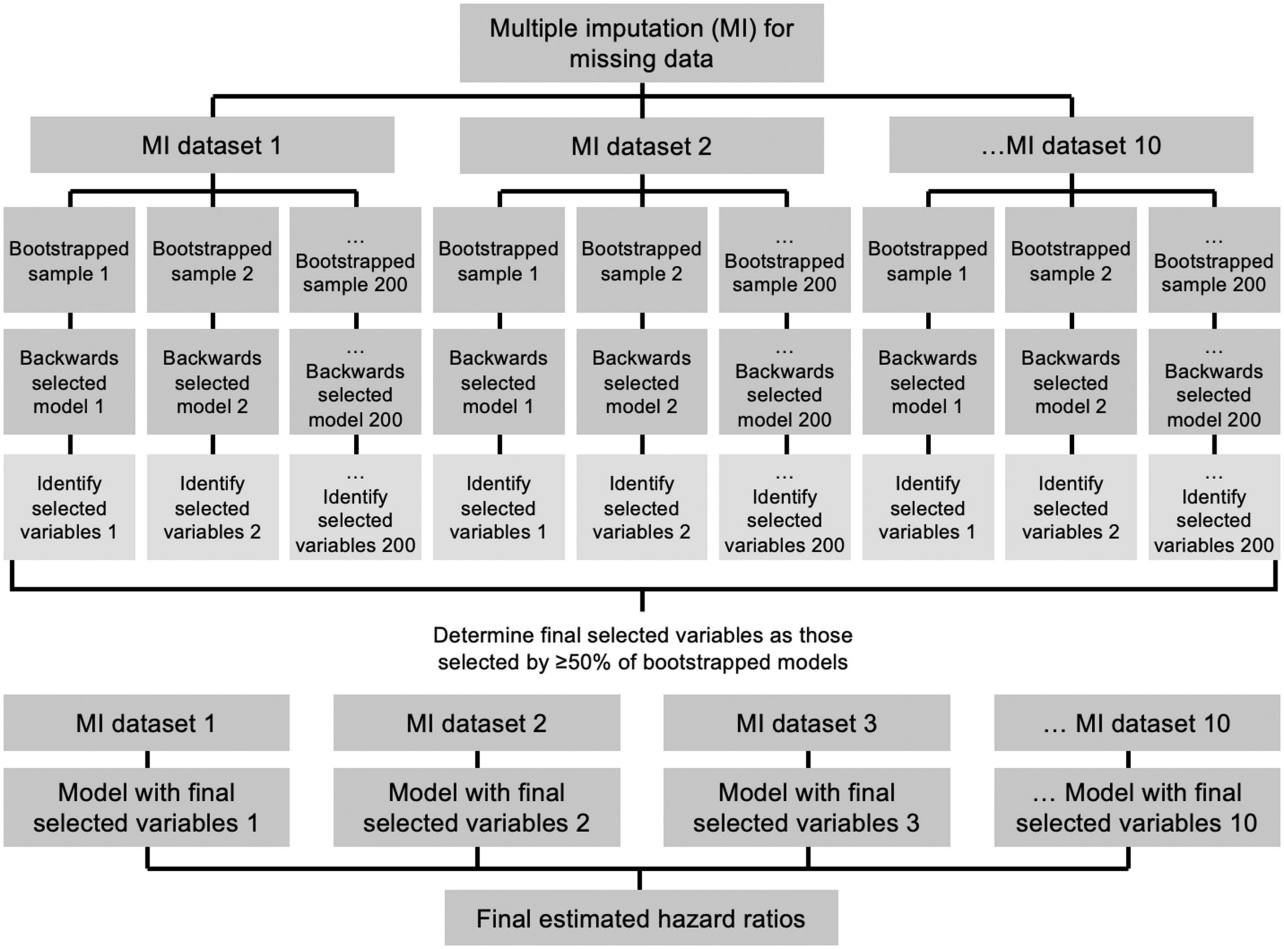
Model ‘voting’ procedure used to determine salient factors

Salient factors were determined using a model ‘voting’ procedure (Figure 1).^12^ Repeated backwards‐selection models of 200 bootstrapped samples were computed to identify factors that minimized the Akaike Information Criterion (AIC).^13^ This process was replicated for each multiply imputed dataset (*m* = 10), resulting in 2000 bootstrapped samples. Factors identified by ≥50% of the multiply imputed, bootstrapped backwards‐selection models were retained in our final analysis. Coefficients were combined using Rubin's rules to correct for uncertainty due to missingness.^14^ Two‐sided significance tests (*α* = .05) were conducted using the median *p*‐value of coefficients estimated for each multiply imputed dataset.^15^ Analyses were performed in Stata 15^11^ and R 4.1.^16^

### 2.6 Ethical considerations

Ethics approval was obtained from Western University's Research Ethics Board (#112986, December 9, 2020) prior to recruitment and updated upon addition of the COVID‐19 sub‐questionnaire. iNPHORM was registered with www.ClinicalTrials.gov (NCT04219514, January 7, 2020).^17^ Ipsos Interactive Services encrypted participant data to ensure confidentiality. Only de‐identified data were transferred to the Western University research team. All participants provided informed consent before enrolment; they could withdraw at any time.

## 3 RESULTS

Among 1206 iNPHORM participants, 817 (mean age: 52.1 [SD: 14.2] years; female: 50.2%) completed the May 2020 questionnaire (with no reported current or previous COVID‐19) and submitted at least one follow‐up thereafter. As of May 2020, 138 (16.89%) reported a diagnosis of type 1 diabetes, and the overall median diabetes duration was 12 (IQR: 15) years (type 1 diabetes: 26 [IQR: 20] years; type 2 diabetes: 11 [IQR: 13] years) (Table 1). All type 1 diabetes participants reported taking insulin (without secretagogues); among participants with type 2 diabetes, 248 (36.5%) reported taking insulin (without secretagogues), 223 (32.8%) secretagogues (without insulin), 155 (22.8%) a combination of insulin and secretagogues and 53 (7.8%) neither insulin nor secretagogues. Ninety‐three percent of follow‐up questionnaires were completed, and 753 (92%) participants remained uncensored prior to study completion. By the end of follow‐up, *n* = 137 (16.8%) received one or more doses of COVID‐19 vaccine (Table S2).

**TABLE 1:** Intrinsic and extrinsic sample characteristics

Characteristic (as reported May 2020)	All participants (*n* = 817)	T1DM (*n* = 138)	T2DM (*n* = 679)
Intrinsic factors			
Age, *n* (%)			
18–29 years	41 (5.02)	21 (15.22)	20 (2.95)
30–49 years	296 (36.23)	64 (46.38)	232 (34.17)
50 years and older	480 (58.75)	53 (38.41)	427 (62.89)
Sex assigned at birth, *n* (%)			
Male	407 (49.82)	50 (36.23)	357 (52.58)
Female	410 (50.18)	88 (63.77)	322 (47.42)
Diabetes type, *n* (%)			
Type 1	138 (16.89)	138 (100)	0
Type 2	679 (83.11)	0	679 (100)
Duration of diabetes (years), median (IQR)	12 (6 to 21)	26 (15 to 35)	11 (5 to 18)
Most recent A1C value, *n* (%)			
Less than or equal to 53 mmol/L (7%)	302 (36.96)	50 (36.23)	252 (37.11)
54–64 mmol/L (7.1–8%)	279 (34.15)	49 (35.51)	230 (33.87)
65–75 mmol/L (8.1–9%)	129 (15.79)	19 (13.77)	110 (16.20)
Greater than or equal to 76 mmol/L (9.1%)	73 (8.94)	18 (13.04)	55 (8.10)
Missing/unknown	34 (4.16)	2 (1.45)	32 (4.71)
BMI, *n* (%)			
BMI less than 30 kg/m^2^	371 (45.41)	107 (77.54)	264 (38.88)
BMI greater than or equal to 30 kg/m^2^	440 (53.86)	31 (22.46)	409 (60.24)
Missing/unknown	6 (0.73)	0	6 (0.88)
Bone disorder, *n* (%)	369 (45.17)	45 (32.61)	324 (47.72)
Missing/unknown	6 (0.73)	1 (0.72)	5 (0.74)
Cancer/HIV, *n* (%)	62 (7.59)	5 (3.62)	57 (8.39)
Missing/unknown	1 (0.12)	0	1 (0.15)
Cardiovascular disease/stroke/hypertension, *n* (%)	503 (61.57)	58 (42.03)	445 (65.54)
Missing/unknown	2 (0.24)	0	2 (0.29)
Chronic kidney disease, *n* (%)	84 (10.28)	11 (7.97)	73 (10.75)
Missing/unknown	11 (1.35)	0	11 (1.62)
Gastrointestinal disease/chronic liver failure, *n* (%)	138 (16.89)	22 (15.94)	116 (17.08)
Missing/unknown	0	0	0
Mental health/eating disorder, *n* (%)	280 (34.27)	46 (33.33)	234 (34.46)
Missing/unknown	5 (0.61)	0	5 (0.74)
Neurological disorder/physical impairment, *n* (%)	226 (27.66)	34 (24.64)	192 (28.28)
Missing/unknown	2 (0.24)	0	2 (0.29)
Respiratory condition, *n* (%)	150 (18.36)	21 (15.22)	129 (19.00)
Missing/unknown	7 (0.86)	1 (0.72)	6 (0.88)
Number of comorbidities^a^, *n* (%)			
0	138 (16.89)	38 (27.54)	100 (14.73)
1	144 (17.63)	28 (20.29)	116 (17.08)
2	154 (18.85)	22 (15.94)	132 (19.44)
3	113 (13.83)	18 (13.04)	95 (13.99)
4	103 (12.61)	13 (9.42)	90 (13.25)
5 or greater	117 (14.32)	13 (9.42)	104 (15.32)
Missing/unknown	48 (5.88)	6 (4.35)	42 (6.19)
Diabetes complication^b^, *n* (%)	497 (60.83)	95 (68.84)	402 (59.20)
One or more severe hypoglycaemia events, *n* (%)	253 (30.97)	59 (42.75)	194 (28.57)
Missing/unknown	3 (0.37)	0	3 (0.44)
Extrinsic factors			
Region, *n* (%)			
Northeast	156 (19.09)	40 (28.99)	116 (17.08)
Midwest	194 (23.75)	33 (23.91)	161 (23.71)
West	142 (17.38)	46 (33.33)	249 (36.67)
South	295 (36.11)	18 (13.04)	124 (18.26)
Missing/unknown	30 (3.67)	1 (0.72)	29 (4.27)
Rurality, *n* (%)			
Urban	230 (28.15)	26 (18.84)	204 (30.04)
Suburban	386 (47.25)	85 (61.59)	301 (44.33)
Rural	201 (24.60)	27 (19.57)	174 (25.63)
Employment, *n* (%)			
Full time	345 (42.23)	65 (47.10)	280 (41.24)
Part time	66 (8.08)	16 (11.59)	50 (7.36)
Unemployed, student, or retired	406 (49.69)	57 (41.30)	349 (51.40)
Highest level of education, *n* (%)			
High school, some high school, or Grade 8	140 (17.14)	26 (18.84)	114 (16.79)
College degree or some college	530 (64.87)	86 (62.32)	444 (65.39)
Degree beyond first college degree	147 (17.99)	26 (18.84)	121 (17.82)
Health literacy^c^, *n* (%)			
Health literate	703 (86.05)	126 (91.30)	577 (84.98)
Somewhat health literate	73 (8.94)	8 (5.80)	65 (9.57)
Not health literate	41 (5.02)	4 (2.90)	37 (5.45)
Income per household member, *n* (%)			
<$15,000	167 (20.44)	36 (26.09)	131 (19.29)
$15,000 to $29,999	269 (32.93)	27 (19.57)	242 (35.64)
$30,000 to $44,999	187 (22.89)	34 (24.64)	153 (22.53)
$45,000 to $59,999	91 (11.14)	14 (10.14)	77 (11.34)
$60,000 to $74,999	46 (5.63)	7 (5.07)	39 (5.74)
$75,000 to $89,999	18 (2.20)	6 (4.35)	12 (1.77)
≥$90,000	31 (3.79)	9 (6.52)	22 (3.24)
Missing/unknown	8 (0.98)	5 (3.62)	3 (0.44)
Number of household members, median (IQR)	2 (2 to 3)	2 (2 to 3)	2 (2 to 3)
Health insurance, *n* (%)			
Private insurance plan	346 (42.35)	76 (55.07)	270 (39.76)
Government‐assistance plan	268 (32.80)	36 (26.09)	232 (34.17)
Multiple insurance plans and other insurance plans	189 (23.13)	22 (15.94)	167 (24.59)
Out‐of‐pocket (i.e. no insurance coverage)	14 (1.71)	4 (2.90)	10 (1.47)
Marital status, *n* (%)			
Never married	176 (21.54)	38 (27.54)	138 (20.32)
Partnered	501 (61.32)	79 (57.25)	422 (62.15)
Divorced, separated, widowed	139 (17.01)	21 (15.22)	118 (17.38)
Missing/unknown	1 (0.12)	0	1 (0.15)
Living arrangement, *n* (%)			
Lives with others	653 (79.93)	116 (84.06)	537 (79.09)
Lives alone	164 (20.07)	22 (15.94)	142 (20.91)
Race, *n* (%)			
White	648 (79.31)	124 (89.86)	524 (77.17)
Non‐white or multiracial	169 (20.69)	14 (10.14)	155 (22.83)
Medication regimen, *n* (%)			
Neither insulin nor secretagogues	53 (6.49)	0	53 (7.81)
Insulin alone	386 (47.25)	138 (100)	248 (36.52)
Secretagogues alone	223 (27.29)	0	223 (32.84)
Insulin and secretagogues	155 (18.97)	0	155 (22.83)
Continuous glucose monitor use, *n* (%)			
No	647 (79.19)	69 (50.00)	578 (85.13)
Yes	166 (20.32)	68 (49.28)	98 (14.43)
Missing/unknown	4 (0.49)	1 (0.72)	3 (0.44)
Number of healthcare visits for diabetes, *n* (%)			
0	474 (58.02)	88 (63.77)	386 (56.85)
1	265 (32.44)	39 (28.26)	226 (33.28)
2	42 (5.14)	7 (5.07)	35 (5.15)
3	16 (1.96)	1 (0.72)	15 (2.21)
4 or more	17 (2.08)	2 (1.45)	15 (2.21)
Missing/unknown	3 (0.37)	1 (0.72)	2 (0.29)
Practices physical distancing, *n* (%)			
Always or often	711 (87.03)	118 (85.51)	593 (87.33)
Sometime, rarely, or never	105 (12.85)	20 (14.49)	85 (12.52)
Missing/unknown	1 (0.12)	0	1 (0.15)

^a^ Comorbidities included bone, joint, or muscle problems; cancer; cardiovascular disease; chronic kidney disease; chronic liver failure; eating disorders; gastrointestinal disease; HIV/AIDS; hypertension; mental health conditions; neurological disorders; and stroke.

^b^ Diabetes complications included amputation, ketoacidosis, foot damage, gastroparesis, hyperosmolar, nephropathy, neuropathy, and retinopathy.

^c^ Health literacy was assessed by asking how often the respondent requires help reading health‐related materials. Respondents were considered health literate if they reported they never or rarely require help. Respondents were considered somewhat health literate if they reported they sometimes require help. Respondents were considered not health literate if they reported they always or often require help.

### 3.1 Incidence proportions of confirmed or probable diagnosis of COVID‐19

From May 2020 to March 2021, 112 of 817 (13.7%) participants reported confirmed (54 [6.6%]) or probable (58 [7.1%]) COVID‐19. Among individuals with type 1 diabetes (*n* = 138), there were 8 (5.8%) confirmed and 10 (7.3%) probable cases. Among those with type 2 diabetes (*n* = 679), there were 46 (6.8%) confirmed and 48 (7.1%) probable cases. The Kaplan–Meier survival curve in Figure S1 displays the probability of remaining COVID‐19 free over the course of follow‐up.

### 3.2 Risk factors of COVID‐19 incidence

Age, BMI, number of chronic comorbidities, most recent A1C, previous severe hypoglycaemia and employment status were identified in ≥50% of backwards‐selected models and retained for analysis (Table 2). Table 3 summarizes the estimated cause‐specific hazard ratios of our final multivariable model. Estimated cause‐specific hazard ratios for COVID‐19 vaccination are reported in Table S3.

**TABLE 2:** Potential risk factors for contracting COVID‐19 considered in the backwards‐selection model

	Proportion of backward‐selected models that retained potential risk factor (%)
Intrinsic factors	
Age	60.15
Sex assigned at birth	47.15
Diabetes type	28.25
Most recent A1C	75.25
BMI	74.05
Bone disorder	25.35
Cancer/HIV	43.85
Cardiovascular disease/stroke/hypertension	22.95
Chronic kidney disease	32.50
Gastrointestinal disease/chronic liver failure	24.55
Mental health/eating disorder	21.20
Neurological disorder/physical impairment	30.10
Respiratory condition	24.65
Number of comorbidities	61.35
Diabetes complication	46.30
One or more severe hypoglycaemia events in the past year	51.00
Extrinsic factors	
Region	32.80
Rurality	36.45
Employment	69.65
Education	48.70
Health literacy	48.45
Income per household member	13.85
Number of household members	31.60
Health insurance	27.70
Marital status	41.85
Living arrangement	33.40
Race	22.10
Medication regimen	34.45
CGM use	36.30
Number of healthcare visits for diabetes	40.00
Practices physical distancing	28.10

*Note:* Shaded potential risk factors were selected by ≥50% of backwards‐selected models and included in the final model. Diabetes duration was dropped due to its collinearity with Age.

**TABLE 3:** Risk factors for acquiring COVID‐19

	Hazard ratio (95% CI)	*p*‐Value
Age		
18 to 29 years	Reference	.33
30 to 49 years	0.57 (0.27 to 1.21)	
50 years and older	0.54 (0.25 to 1.17)	
Employment		
Full time	Reference	.07
Part time	1.39 (0.71 to 2.70)	
Unemployed, student or retired	0.68 (0.43 to 1.08)	
BMI greater than or equal to 30 kg/m^2^	1.63 (1.05 to 2.52)	.02
Most recent A1C value, *n* (%)		
Less than or equal to 53 mmol/L (7%)	Reference	.06
54 to 64 mmol/L (7.1–8%)	0.61 (0.38 to 1.00)	
65 to 75 mmol/L (8.1–9%)	0.65 (0.34 to 1.26)	
Greater than or equal to 76 mmol/L (9.1%)	1.27 (0.70 to 2.29)	
Number of comorbidities	1.16 (1.05 to 1.27)	.002
One or more severe hypoglycaemia events in the past year	0.82 (0.41 to 1.65)	.57

Reported COVID‐19 incidence between May 2020 and March 2021 statistically significantly increased with presence of obesity (BMI≥30 kg/m^2^ vs. <30 kg/m^2^) (HR 1.63 [1.05; 2.52]_95% CI_, *p* = .02) and number of chronic comorbidities (HR 1.16 [1.05; 1.27]_95% CI_, *p* = .002). A marginally statistically significant effect was observed for overall A1C (*p* = .06). Specifically, rates of COVID‐19 were higher for A1C values ≤53.0 mmol/L (≤7%) versus 54–64 mmol/L (7.1%–8%) (HR 0.61 [0.38; 1.00]_95% CI_) or 65–75 mmol/L (8%–9.1%) (HR 0.65 [0.34; 1.26]_95% CI_), but lower when compared to values ≥76 mmol/L (≥9.1%) (HR 1.27 [0.70; 2.29]_95% CI_). Overall, employment status also marginally significantly affected COVID‐19 incidence (*p* = .07): part‐time workers were more likely to report COVID‐19 than full‐time workers (HR 1.39 [0.71; 2.70]_95% CI_), but less likely than those who were unemployed, retired or students (HR 0.68 [0.43; 1.08]_95% CI_).

Negative, non‐significant associations were detected for previous severe hypoglycaemia (HR 0.82 [0.41; 1.65]_95% CI_) and pairwise comparisons of individuals ≥50 versus 18–29 years old (HR 0.54 [0.25; 1.17]_95% CI_).

## 4 DISCUSSION

Americans with type 1 and 2 diabetes with COVID‐19 exhibit higher rates of morbidity,^3^ mortality,^3^ and long COVID than people without diabetes.^4^ COVID‐19 prevention strategies optimally tailored to this clinically vulnerable population are imperative. Nevertheless, while research on hospital‐based severe outcomes and treatment advances,^5^ virtually no diabetes‐specific studies exist on how to thwart infection in the first place.^18^

This primary epidemiologic investigation is the first to comprehensively quantify the real‐world, time‐varying predictors of COVID‐19 in the general US population with diabetes. Between May 2020 and March 2021, 112 of 817 (13.7%) participants in our study reported either a confirmed or probable diagnosis of COVID‐19. In total, six risk factors were selected in our final model: age, obesity (BMI ≥ 30 kg/m^2^), increased number of chronic comorbidities, most recent A1C, previous severe hypoglycaemia, and employment status. Only obesity and comorbidity achieved statistical significance.

Saliently, COVID‐19 rates were 63% higher in respondents with a BMI ≥30 kg/m^2^ versus <30 kg/m^2^ (*p =* .02). Obesity has been identified as the most prevalent and predictive comorbidity of severe outcomes among hospitalized COVID‐19 cases with diabetes.^18^ We now provide new evidence that it may also augment SARS‐CoV‐2 initiation.^19^ In people with obesity, low‐level chronic inflammation can aggravate viral susceptibility by reducing macrophage activation, proinflammatory cytokine production, and B‐ and T‐cell responses.^20^ Adipose tissue can also act as a major source of inflammatory molecules, including interleukin‐6 (IL‐6), a gene implicated in the expression of angiotensin converting enzyme 2 (ACE2) and SARS‐CoV‐2.^19^ With >85% of Americans with diabetes diagnosed as overweight/obese, our analysis signals a key opportunity for effective pandemic surveillance and risk mitigation.^21^

But even more generally, we found that chronic diabetes comorbidity, irrespective of obesity, can promote COVID‐19, suggesting the need for a broad scope prevention plan. In the US, over 80% of people with diabetes have at least one other underlying health condition.^22^ Results of our time‐varying analysis revealed a 16% increased rate of COVID‐19 for each additional comorbidity reported (*p* = .002). This finding expands on previous hospital‐based analyses reporting worse COVID‐19 outcomes in people with diabetes and coexisting conditions versus diabetes alone.^18^ Increased inflammation, immunosuppression and cardio‐renal impairments may underpin the predictive role of comorbidity on SARS‐CoV‐2 susceptibility.^23^ Pathophysiologic research has also uncovered distinct mechanisms for ACE2 expression in people with coexisting conditions that may modulate SARS‐CoV‐2 entry into lung cells. In particular, comorbid diabetes has been shown to propagate IL‐6 and INS gene expression, leading to increased ACE2 via NAD‐dependent histone deacetylase Sirtuin.^24^

The role of glycaemia on COVID‐19 susceptibility has been widely debated. In our study, haemoglobin A1C—a well‐established marker of glycaemic control—was identified as a marginally significant risk factor of COVID‐19 (*p* = .06). Participants with A1C values ≥76 mmol/L (≥9.1%) reported 27% higher COVID‐19 rates than those with values ≤53.0 mmol/L (≤7%). Thus, in addition to worsening COVID‐19 severity and fatality, elevated plasma glucose levels may also trigger SARS‐CoV‐2 binding and viral replication.^5^, ^25^ However, we observed a 35%–39% lower rate of COVID‐19 in people with A1Cs of 54–75 mmol/L (7.1–9.1%) versus ≤53.0 mmol/L (≤7%). It is plausible this effect is mediated by increased hypoglycaemia in people with tightly controlled diabetes (A1C ≤53.0 mmol/L [≤7%]).^26^ Recent data out of Scotland demonstrated greater COVID‐19 morbidity and mortality risk in people exposed to frequent low blood glucose.^27^ As hypoglycaemia is known to induce inflammation, including IL‐6 expression, and decrease immune responsiveness, it is not surprising that it may also exacerbate biologic predisposition to infection.^25^ Amid challenges to sustain routine diabetes care during the pandemic,^28^ it is essential that efforts to optimize glucose management not wane.

Interestingly, most socio‐demographic factors (e.g. race) were not retained in our final model—perhaps due to insufficient power, or proximate clinical variables nullifying upstream effects. Employment status emerged as the only marginally significant extrinsic risk factor in our study (*p* = .07). Relative to full‐time workers, COVID‐19 rates were 39% higher in part‐time workers, yet 32% lower in unemployed participants, retirees and students. Employment‐related environmental and behavioural dynamics could conceivably influence the probability of SARS‐CoV‐2 contact.^24^ For example, part‐time work (often on‐site and involving close physical proximity),^29^ in contrast to at‐home (un)employment, may not only exacerbate environmental exposure, but also inhibit optimal prevention behaviour.^29^ Clinical and public health strategies should account for the possible interplay between employment status and risk for COVID‐19.^24^

Several additional variables were included in the analysis but failed to reach statistical significance. This could be due to a lack of association between these variables and susceptibility to COVID‐19, or because our analysis was inadequately powered to identify these associations due to its smaller sample size. Despite a strong magnitude of effect (HR 0.82 [0.41 to 1.65]_95% CI_), previous severe hypoglycaemia events were not found to independently predict COVID‐19 rates, possibly due to the collinear effects of glycaemic control. Additionally, like previous population‐based studies, COVID‐19 rates did not differ by diabetes type.^5^ Age was, overall, statistically insignificant; though, pairwise comparisons suggested a ~ 50% lower rate of COVID‐19 in older (≥50 years) versus younger (18–29 years) participants, echoing previous hospital‐based data.^27^

Targeted COVID‐19 vaccine and prevention strategies are crucial to protecting people with diabetes from potentially devasting outcomes. Our study identifies diabetes groups at highest risk of COVID‐19 contraction, providing an evidence‐based roadmap for effective and efficient risk management and outbreak control.

### 4.1 Strengths and limitations

This iNPHORM sub‐analysis examines a large general cohort of American adults with type 1 or 2 diabetes recruited from a probability‐based, real‐world internet panel with high participation rates and minimal loss‐to‐follow‐up. Our primary epidemiologic investigation contrasts the retrospective, hospital‐based research currently dominating the diabetes COVID‐19 evidence base. In our study, both confirmed and probable COVID‐19 cases were assessed, illuminating community‐level pathways to infection prevention in diabetes.

Epidemiologic features of COVID‐19 were quantified in a broad sample of cases and non‐cases; plausible intrinsic and extrinsic variables were selected a priori. Continuous and repeated monitoring enabled assessment of time‐varying exposures on SARS‐CoV‐2 incidence leading to COVID‐19 between May 2020 and March 2021, and effect directions yet discerned by the prevailing literature (e.g. impact of glycaemic control on COVID‐19).

Questionnaires and data collection were standardized over follow‐up. The use of online survey modes with email optimized reach, accessibility, respondent honesty and representativeness of data capture.^30^ De‐identified participant information was collected to reduce social desirability bias.

Some limitations should be noted. Selection biases may have arisen to the extent that study respondents differed non‐randomly from the general US population. Coverage bias may limit study generalizability. For example, in addition to restrictions on eligible medication regimens, our sample comprised mostly white, educated, and insured participants. Over‐representation of these subgroups may have biased the effect of certain risk factors, particularly extrinsic determinants, towards the null. Lastly, volunteer biases may have influenced COVID‐19 characterization in our study; though, in what direction is uncertain. By extension, survivorship bias cannot be discounted.

We did not assess people without diabetes, so excess risk of COVID‐19 attributable to diabetes could not be calculated. Further, while we could estimate the incidence of COVID‐19 across our study sample, we could not distinguish COVID‐19 cases requiring hospitalization or resulting in increased morbidity or mortality. Self‐reported COVID‐19 status and other risk measurements (e.g. A1C) may have been biased by errors in recall. Our model does not represent possible SARS‐CoV‐2 reinfection resulting in confirmed or probable COVID‐19. Finally, SARS‐CoV‐2 latency beyond the observation period and uncounted asymptomatic cases may have led to conservative risk coefficients. As a corollary to this, it is possible that factors identified in our study may relate more with symptomatic illness resulting from SARS‐CoV‐2 infection, and the possible increased likelihood of COVID‐19 diagnosis. Continued diabetes research is required to monitor waning antibody levels, immune evasion, and future variants that can affect diabetes population susceptibility over time.

## 5 CONCLUSION

Leveraging iNPHORM data between May 2020 and March 2021, we present the first prospective, longitudinal epidemiologic analysis of community‐based COVID‐19 incidence and time‐varying risk factors (intrinsic and extrinsic) in the general US public with type 1 or 2 diabetes. Our real‐world results indicate that vaccination rollouts and other outbreak strategies should prioritize Americans with diabetes reporting a BMI≥30 kg/m^2^, a concomitant health condition, A1C values ≥9.1% and ≤7%, or part‐time employment.

Persistent waves of infection and patterns of endemicity threaten to destabilize our path to pandemic recovery. Measures to protect against SARS‐CoV‐2 must remain at the forefront of all healthcare and policy decision‐making, especially among those most vulnerable to infection. Our study unveils promising signposts to mitigate the severe effects of COVID‐19 in diabetes and associated long‐term health burden.

## AUTHOR CONTRIBUTIONS

**Alexandria Ratzki‐Leewing:** Conceptualization (equal); data curation (equal); investigation (equal); methodology (supporting); project administration (equal); writing – original draft (lead); writing – review and editing (lead). **Jason E. Black:** Data curation (lead); formal analysis (lead); methodology (lead); software (equal); writing – review and editing (equal). **Bridget L. Ryan:** Conceptualization (equal); investigation (equal); project administration (equal); supervision (equal). **Stewart B. Harris:** Conceptualization (equal); investigation (equal); project administration (equal); supervision (equal).

## CONFLICT OF INTEREST

A. R.‐L.: Sanofi: grant, fees paid for presentations; Eli Lilly: consultant, fees paid for presentations; Novo Nordisk: consultant.; J.E.B.: Nothing to disclose.; B.L.R.: Nothing to disclose.; S.B.H: Sanofi: grant, member advisory board, consultant; Eli Lilly: grant, member advisory board, consultant, clinical studies; Novo Nordisk: grant, member advisory board, consultant, clinical studies; Janssen: grant, member advisory board, consultant; AstraZeneca: grant, member advisory board, consultant, clinical studies; Abbott: grant, member advisory board, consultant; Boehringer Ingelheim: grant, member advisory board, consultant, clinical studies; JDRF: grant; Lawson: grant; Canadian Institutes of Health and Research: grants.

## Supporting information


Appendix S1

## Data Availability

Research data are not shared.
