# Supplementary material for: Real‐world risk factors of confirmed or probable COVID‐19 in Americans with diabetes: A prospective, community‐based study (iNPHORM)
Source: Endocrinol Diabetes Metab. 2022 May 29;5(4):e342. doi: 10.1002/edm2.342 (PMC9258990; doi:10.1002/edm2.342)
Supplement: Supplementary file 1 — Appendix S1 [file EDM2-5-0-s001.docx]

**Supplementary Appendix**

Screener/Baseline Questionnaire Measures

At screener, we collected data on age, sex assigned at birth, geographic region, diabetes type, and diabetes medication regimen (insulin and/or secretagogues). At baseline, we collected data on socio-demographic (i.e., rurality, employment, highest level of education, health literacy, income, health insurance, marital status, living arrangement, and race); clinical (i.e., diabetes duration, most recent A1C, body mass index, use of glucose monitoring, frequency of diabetes healthcare, chronic comorbidity, diabetes complications, and past severe hypoglycaemia incidence); and behavioural (i.e., COVID-19-related physical distancing) variables.

Follow-up Questionnaire Measures

Follow-up questionnaires collected data on self-reported incidences of severe hypoglycemia (since the last completed questionnaire), as well as information on participants’ recent clinical care and health. At Months 4, 8 and 12 we obtained updated information on employment, household income, and health insurance.

**SUPPLEMENTAL TABLES**

**Table S1**. Description of prognostic factors considered by backwards selection model

| **Prognostic variable** | **Recall time frame** | **Value can change over time** | **Measurement unit(s)/response categories**^†^ |
| --- | --- | --- | --- |
| *Intrinsic factors* | | | |
| Age | Current | No | 18 to 29 years; 30 to 49 years; 50 years and older |
| Sex assigned at birth | Assigned at birth | No | Male; Female |
| Type of diabetes | Current | No | Type 1 diabetes; Type 2 diabetes |
| HbA1C value | Most recent | Yes | Less than or equal to 7%; 7.1% to 8%; 8.1% to 9%; Greater than or equal to 9.1%; Unsure |
| BMI | Current | No | Less than 30 kg/m^2^; Greater than or equal to 30 kg/m^2^ |
| Bone, joint, or muscle problem | In lifetime | No | No; Yes; Unsure |
| Cancer or HIV/AIDS | In lifetime | No | No; Yes; Unsure |
| Cardiovascular condition, Stroke or transient ischemic attack, or Hypertension | In lifetime | No | No; Yes; Unsure |
| Chronic kidney disease | In lifetime | No | No; Yes; Unsure |
| Gastrointestinal disease or Chronic liver failure or liver disease | In lifetime | No | No; Yes; Unsure |
| Mental health condition or Eating disorder | In lifetime | No | No; Yes; Unsure |
| Neurological disorder or Physical impairment | In lifetime | No | No; Yes; Unsure |
| Respiratory condition | In lifetime | No | No; Yes; Unsure |
| Number of chronic co-morbidities | In lifetime | No | Bone, joint, or muscle problem; Cancer; Cardiovascular condition; Chronic kidney disease; Chronic liver failure or liver disease; Eating disorder; Gastrointestinal disease; HIV/AIDS; Hypertension; Mental health condition; Neurological disorder; Physical impairment; Respiratory condition; Stroke or transient ischemic attack |
| Diabetes-related complications | In lifetime | Yes | Amputation of toes, feet, or legs; Diabetic ketoacidosis; Foot damage; Gastroparesis; Hyperosmolar hyperglycemic nonketotic coma; Nephropathy; Neuropathy; Retinopathy |
| One or more daytime and nocturnal severe hypoglycemia event | Past 12 months or since last iNPHORM questionnaire was completed | Yes | No; Yes; Unsure |
| *Extrinsic factors* | | | |
| Region | Current | No | Northeast (Connecticut, Maine, Massachusetts, New Hampshire, Rhode Island, Vermont, New Jersey, New York, and Pennsylvania); Midwest (Illinois, Indiana, Michigan, Ohio, Wisconsin, Iowa, Kansas, Minnesota, Missouri, Nebraska, North Dakota, and South Dakota); South (Delaware, Florida, Georgia, Maryland, North Carolina, South Carolina, Virginia, Washington, D.C., West Virginia, Alabama, Kentucky, Mississippi, Tennessee, Arkansas, Louisiana, Oklahoma, and Texas); West (Arizona, Colorado, Idaho, Montana, Nevada, New Mexico, Utah, Wyoming, Alaska, California, Hawaii, Oregon, and Washington) |
| Rurality | Current | No | Urban; Suburban; Rural |
| Employment | Current | Yes | Working full-time, including self-employment (25 hours per week or more); Working part-time, including self-employment (less than 25 hours per week); Unemployed, student, or retired (Temporarily laid off; Temporarily unemployed due to a health-related reason; Unemployed and looking for work; Unemployed and not looking for work; Unable to work due to disability; Going to school; Looking after house/family; Retired) |
| Highest level of education | Current | No | Highschool, some high school, or Grade 8 (Grades 1 through 8; Grades 9 through 12, no diploma; Regular high school diploma or GED/alternative credential); College degree or some college; Degree beyond completing first college Bachelor’s degree |
| Health literacy | Current | No | Modified BRIEF: Health Literacy Screening Tool (1) 3-item survey |
| Income per household member (before taxes and deductions) | Past 12 months | Yes | <$15,000; $15,000 to $29,999 ; $30,000 to $44,999; $45,000 to $59,999; $60,000 to $74,999; $75,000 to $89,999; ≥$90,000 |
| Number of people in household | Current | No | 1; 2; 3; 4; 5; 6; 7; 8; 9; 10 or more |
| Health insurance | Current | Yes | Private insurance plan (Insurance through a current or former employer or union [of you or another family member] that is not a high deductible plan, Insurance purchased directly from an insurance company that is not a high deductible plan, High deductible plan); Government-assistance plan (Medicare, Medicaid, Medical Assistance, or any kind of government-assistance plan, TRICARE, Veterans Affairs, Native American Health Service, Consolidated Omnibus Budget Reconciliation Act (COBRA) insurance); Multiple insurance plans (Private insurance plan and Government-assistance plan) or other insurance plans; Out-of-pocket |
| Marital status | Current | No | Never married; Partnered (Married; domestic partnership); Divorced, widowed, or separated |
| Living arrangement | Current | No | Lives with others (Lives with a spouse or partner; Lives with minor children; Lives with other adult family members; Lives with other people); Lives alone |
| Race | Current | No | White; Non-white or multiracial (Black or African American; Asian; Hispanic, Latino/a, or Spanish origin; Native Hawaiian or other Pacific Islander; American Indian or Alaska Native; Other or Multiracial) |
| Use of insulin and/or secretagogues | Current | Yes | Insulin alone; Secretagogues alone; Insulin and secretagogues; and Neither insulin or secretagogues |
| Continuous glucose monitoring device use | In lifetime | Yes | No; Yes; Unsure |
| Number of healthcare visits for diabetes | Past 12 months or since last iNPHORM questionnaire was completed | Yes | Frequency |
| Social/physical distancing or shelter-in-place | Since last iNPHORM questionnaire was completed | Yes | Always or Often; Sometimes, Rarely, or Never |
| (1) Haun, J., Luther, S., Dodd, V., & Donaldson, P. (2012). Measurement variation across health literacy assessments: implications for assessment selection in research and practice. J Health Commu,17 Suppl 3:141-59. Doi: 10.1080/10810730.2012.712615.  BMI, body mass index  † Response categories may differ from actual questionnaire | | | |

Table S2 – COVID-19 vaccination rates in study population

|  | All respondents (n=817) | T1DM  (n=138) | T2DM  (n=679) |
| --- | --- | --- | --- |
| One or two doses of COVID-19 vaccine | 137 (16.8%) | 31 (22.5%) | 106 (15.6%) |

Table S3 – Cause-specific hazard ratios for COVID-19 vaccination

|  | **Hazard ratio (95% CI)** | ***p*-value** |
| --- | --- | --- |
| Age |  | 0.049 |
| 18 to 29 years | Reference |  |
| 30 to 49 years | 1.21 (0.55 to 2.66) |  |
| 50 years and older | 1.97 (0.89 to 4.36) |  |
| Employment |  | 0.10 |
| Full time | Reference |  |
| Part time | 1.01 (0.44 to 2.32) |  |
| Unemployed, student, or retired | 0.95 (0.61 to 1.47) |  |
| BMI greater than or equal to 30 kg/m^2^ | 0.84 (0.57 to 1.23) | 0.39 |
| Most recent A1C value, n (%) |  | 0.03 |
| Less than or equal to 53 mmol/L (7%) | Reference |  |
| 54 to 64 mmol/L (7.1-8%) | 0.62 (0.40 to 0.96) |  |
| 65 to 75 mmol/L (8.1-9%) | 0.44 (0.20 to 0.98) |  |
| Greater than or equal to 76 mmol/L (9.1%) | 0.81 (0.36 to 1.82) |  |
| Number of comorbidities | 0.98 (0.88 to 1.10) | 0.77 |
| One or more severe hypoglycemia events in the past year | 0.55 (0.21 to 1.40) | 0.20 |

**SUPPLEMENTAL FIGURES**

Figure S1 – Kaplan Meier survival curve for confirmed or probable diagnosis of COVID-19
